# The Role of Catechins in Cellular Responses to Oxidative Stress

**DOI:** 10.3390/molecules23040965

**Published:** 2018-04-20

**Authors:** Jurga Bernatoniene, Dalia Marija Kopustinskiene

**Affiliations:** 1 Department of Drug Technology and Social Pharmacy, Medical Academy, Lithuanian University of Health Sciences, Eiveniu 4, LT-50161 Kaunas, Lithuania; 2 Institute of Pharmaceutical Technologies, Medical Academy, Lithuanian University of Health Sciences, Eiveniu 4, LT-50161 Kaunas, Lithuania; daliamarija.kopustinskiene@lsmuni.lt

**Keywords:** catechin, ROS, cancer, cardiovascular diseases, neurodegenerative disorders

## Abstract

Catechins are polyphenolic compounds—flavanols of the flavonoid family found in a variety of plants. Green tea, wine and cocoa-based products are the main dietary sources of these flavanols. Catechins have potent antioxidant properties, although in some cases they may act in the cell as pro-oxidants. Catechins are reactive oxygen species (ROS) scavengers and metal ion chelators, whereas their indirect antioxidant activities comprise induction of antioxidant enzymes, inhibition of pro-oxidant enzymes, and production of the phase II detoxification enzymes and antioxidant enzymes. Oxidative stress and ROS are implicated in aging and related dysfunctions, such as neurodegenerative disease, cancer, cardiovascular diseases, and diabetes. Due to their antioxidant properties, catechins may be beneficial in preventing and protecting against diseases caused by oxidative stress. This article reviews the biochemical properties of catechins, their antioxidant activity, and the mechanisms of action involved in the prevention of oxidative stress-caused diseases.

## 1. Introduction

Catechins are natural polyphenolic compounds—flavan-3-ols (or flavanols), belonging to the flavonoid family. They are found in abundant concentrations in a variety of fruits, vegetables and plant-based beverages. The name catechin is derived from Cutch tree (*Acacia catechu* L.f.) [1]. High concentrations of catechin can be found in fresh tea leaves, rock-rose leaves, broad beans, red wine, black grapes, strawberries, and apricots [2]. Apples, blackberries, broad beans, cherries, black grapes, pears, raspberries, and chocolate are rich in epicatechin [2]. The main dietary source of catechins is green tea [1]. Recently, the monograph of green tea was included in the European Pharmacopoeia, where green tea is standardized for caffeine content (min 1.5%) and for total content of catechins, expressed as (−)-epigallocatechin-3-*O*-gallate (min 8%) [3]. Catechin, epicatechin, and gallate epicatechin are present in red wine where reacting with tannins they are responsible for wine flavor [4]. Cocoa based products such as chocolate also contain catechin and epicatechin in high amounts [5].

## 2. Chemical Properties of Catechins

**Figure 1:**
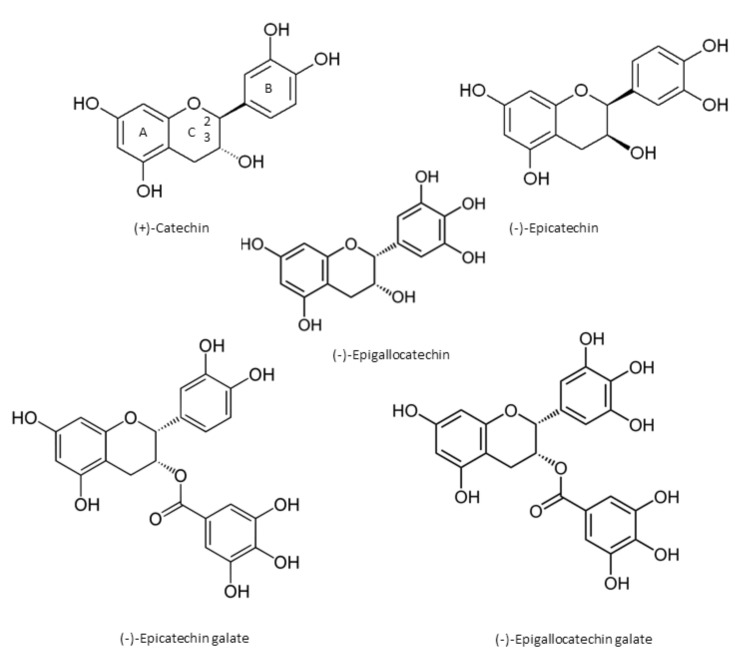
The main chemical structures of catechins.

Catechin chemically consists of two benzene rings (A- and B-rings) and a dihydropyran heterocycle (the C-ring) with a hydroxyl group on carbon 3 [1]. There are two chiral centers on the molecule on carbons 2 and 3. Catechin stereoisomers in *cis* ((−)-epicatechin) or *trans* ((+)-catechin) configuration, with respect to carbons 2 and 3, are flavan-3-ol compounds [1,6,7]. Through esterification with gallate groups, flavanols can form gallic acid conjugates epicatechin gallate (ECG), epigallocatechin (EGC), and epigallocatechin gallate (EGCG) [1,6,7] (Figure 1). Condensed catechins are obtained via catechin polymerization [1]. The most common oligomers derived from epicatechin are A-type and B-type procyanidins. In A-type dimers, the monomers are linked by both a 4→8 carbon–carbon and a 2→O7 ether bond, and the monomers of the B-type dimers are linked through 4→8 carbon–carbon bonds [1].

## 3. Catechins as Antioxidants

Catechins appear to be able both to generate and to scavenge free radicals and show their beneficial effects due to a combination of both mechanisms [8,9].

**Figure 2:**
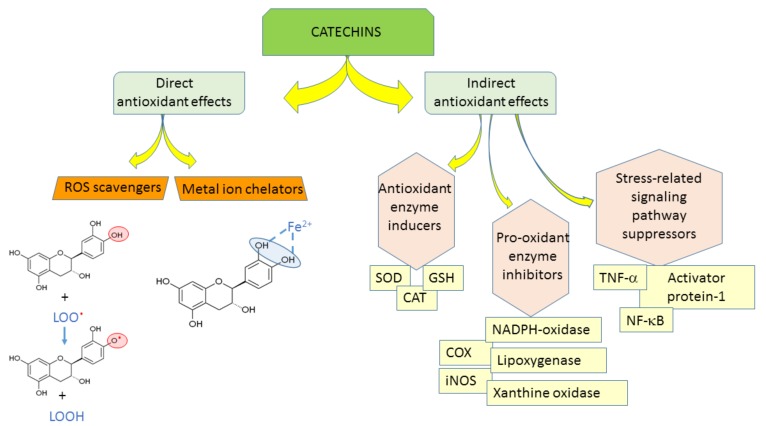
Antioxidant properties of catechins. ROS-reactive oxygen species, SOD-superoxide dismutase, CAT-catalase, GSH-glutathione peroxidase, NADPH-oxidase-nicotinamide adenine dinucleotide phosphate oxidase, COX-cyclooxygenase, iNOS-inducible nitric oxide synthase, TNF-α–tumor necrosis factor alpha, NF-κB-nuclear factor kappa-light-chain-enhancer of activated B cells.

The antioxidant efficacy of catechins is exerted through (1) direct mechanisms—scavenging ROS, chelating metal ions; and (2) indirect mechanisms—inducing antioxidant enzymes, inhibiting pro-oxidant enzymes, and producing phase II detoxification enzymes and antioxidant enzymes (Figure 2) [10]. Catechin and its diastereoisomers all have common chemical structures—phenolic hydroxyl groups that are able to stabilize the free radicals [11]. This property is responsible for their direct antioxidant activities where catechins can act as free radical scavengers. Phenolic hydroxyl groups of catechins can react with reactive oxygen and reactive nitrogen species in a termination reaction, which breaks the cycle of generation of new radicals (Figure 2). Catechins donate one electron of phenolic OH group, thus reducing free radicals and the aromatic group maintains stability through the resonance of the resultant aroxyl radicals [12,13]. Following interaction with the initial reactive species, a radical form of the antioxidant is produced, which is stabilized by charge delocalization caused by the interaction of the phenolic hydroxyl groups with the π-electrons of the benzene ring [14]. The antioxidant potential of phenolic compounds depends on the number and arrangement of the hydroxyl groups and the extent of structure conjugation [15,16]. The number of hydroxyl groups in the molecule correlates positively with the antioxidant activity of phenolic compounds [15]. The relative hierarchy of effectiveness of catechins as radical scavengers is EGCG > ECG > EGC > EC > C [15,17,18,19,20]. As free radical scavengers, catechins are able to stop radical chain reactions preventing cellular lipids from oxidation.

The antioxidant capacity of phenolic compounds is also attributed to their ability to chelate metal ions involved in the production of free radicals [11]. Adjacent hydroxyl groups in the molecule can act as iron chelation sites (Figure 2).

As indirect antioxidants, catechins regulate protein synthesis and signaling pathways [12]. In addition, catechins can up-regulate anti-oxidant enzymes [21,22]. Mice given 0.2% catechins in drinking water showed significantly increased activities of superoxide dismutase (SOD), catalase (CAT), and glutathione peroxidase (GSH) [23], which play key roles in scavenging ROS. Green tea consumption for two weeks induced the expression of catalase in aorta of spontaneously hypertensive rats [24]. In addition, catechins complemented the functions of glutathione participating in vitamin E recycling [25]. Furthermore, green tea increased the plasma and tissue glutathione levels in several animal studies [7,26,27,28].

Catechins can inhibit prooxidant enzymes, e.g., NADPH (nicotinamide adenine dinucleotide phosphate)-oxidase, or modulate interaction of ligands with receptors, e.g., tumor necrosis factor alpha (TNF-α) [12], also, they can suppress many oxidative stress-related pathways responsible for the inflammation processes [12]. Catechins modulate the activities of redox-sensitive transcription factors-nuclear factor kappa-light-chain-enhancer of activated B cells (NF-κB) and activator protein-1 (AP-1), which are very important in the response to pathogenesis-related oxidative stress [1].

Catechins can interact with membranes via adsorption or penetration into the lipid bilayers [11]. Phenolic structures often have the potential to strongly interact with proteins due to interaction of their hydrophobic benzene rings with protein proline residues and hydrogen-bonding potential of the phenolic hydroxyl groups [11]. Furthermore, as catechins are structurally similar to ATP, they could competitively bind to the enzyme ATP-binding sites. Structural/conformational properties and hydrogen bonding are also suggested as mechanisms for catechin interactions with transcriptional factors [11].

## 4. Catechins in Oxidative Stress-Caused Diseases

Inflammation and oxidative stress are considered to be major causes of various chronic disturbances [29,30]. Several age-associated diseases such as cancer, neurodegenerative diseases—Parkinson’s disease, Alzheimer’s disease, cardiovascular diseases, and diabetes are linked to changes in oxidant-antioxidant balances and free radical damage [1,31]. Due to their antioxidant properties, catechins may be beneficial in preventing and protecting from pathologies caused by oxidative stress [1,18].

### 4.1. Cardiovascular Diseases

Oxidative stress is implicated in the progression of various cardiovascular diseases, including hypertension, endothelial dysfunction, atherosclerosis, ischemic heart diseases, cardiomyopathy, cardiac hypertrophy, and congestive heart failure [32]. ROS-caused ischemia/reperfusion injury plays an essential role in myocardial damage during ischemic stroke and myocardial infarction [33]. The causes of cardiovascular diseases are multifactorial, also involving abnormalities in lipid metabolism and disturbances in vascular cells [32].

Catechins from green tea decreased blood pressure and reduced the risk of stroke and coronary heart disease [34,35]. Catechins alleviated conditions associated with vascular dysfunction, including vascular inflammation and smooth muscle cell proliferation, blood platelet aggregation, lipoprotein oxidation, altered lipid profile, and vascular reactivity [7]. Catechins play a crucial role in the balance of vasoconstricting substances, such as endothelin-1, prostaglandins, angiotensin II, and vasodilating substances, such as nitric oxide, prostacyclin and various endothelium-derived hyperpolarizing factors [36,37]. Catechins inhibited oxidative damage and reduce lipid peroxidation in vascular smooth muscle cells [38]. Crude extract from species of the *Cistus* genus—*Cistus incanus* L. and *Cistus monspeliensis* L., rich in catechins [39,40,41], showed potent free radical scavenging activity, protecting DNA from cleavage and inhibited lipid peroxidation in rat liver microsomes [42]. Epigallocatechin gallate enhanced expression of p53, p21, and NF-κB, induced the apoptosis of vascular smooth muscle cells and prevented the development of atherosclerosis [43,44]. In addition, catechins reduced the accumulation of cholesterol and its oxidation products in artery walls when it was combined with free radicals in vivo, thus improving blood circulation [45]. Catechins could normalize the levels of cholesterol, reducing the blood fat deposition [46]. Furthermore, catechins reduced the expression of cytokines, NF-κB, intercellular adhesion molecule 1 (ICAM-1), and TNF-α responsible for inflammation, and suppressed myocardial inflammation in rats [32,46].

Animal model studies suggested that green tea bioactive components might protect against the development of coronary heart disease by reducing blood glucose levels and body weight [1,34]. Green tea flavonoids had insulin-like activities as well as insulin-enhancing activity. Epigallocatechin galate inhibited intestinal glucose uptake by the sodium-dependent glucose transporter (SGLT1), indicating its increase in controlling blood sugar [47].

### 4.2. Cancer

The catechins present in tea leaves inhibited the growth of cancer cells [48]. Furthermore, catechins could possess the anti-carcinogenic activity in many experimental systems and in many kinds of organs, including lung, liver, pancreas, esophagus, stomach, small intestine, colon, bladder, skin, the oral cavity, prostate and mammary gland [49,50,51,52,53]. It has been proved that catechins could inhibit carcinogenesis, tumor growth, cancer cell invasion, and tumor angiogenesis, by suppressing the induction of proangiogenic factors [12,54]. In xenograft models, green tea catechins inhibited tumour growth and suppressed metastasis of metastasis specific mouse mammary carcinoma 4T1 cells [55] and reduced tumor blood vessel formation in estrogen receptor-negative breast cancer [56]. Experimental studies demonstrated that anti-cancer activity likely resulted from the antioxidant activity and the direct binding of green tea polyphenols to proteins, thus modulating multiple cellular signaling pathways [57]. Regulation of the apoptotic process is a critical step in the prevention or treatment of cancer. Apoptosis is known to play a significant role in eliminating precancerous and cancer cells and function as a protective mechanism [58,59]. Catechins, especially epigallocatechin galate, induced apoptosis and cell-cycle arrest, inhibited NF-κB, and suppressed cyclooxygenase-2 (COX) overexpression in vitro and in animal models [58]. Catechins also modulated apoptosis by altering the expression of anti- and proapoptotic genes [60,61,62]. The effects of catechins on apoptosis depended on their concentration used for the studies. Low concentrations (1 µM) induced immediate expression of antiapoptotic bcl-xL and/or bcl-2, whereas Bax expression was reduced [63,64,65]. A proapoptotic pattern of gene expression—upregulation of caspases-3 and 10, Fas and the Fas ligand, the NF-κB p105 subunit, and p53 was observed at high concentrations (50 µM) of catechins [1,66].

### 4.3. Neurodegenerative Diseases

Oxidative stress and ROS are implicated in aging and related neurodegenerative disorders [67,68]. Oxidative stress also plays a crucial role in cerebral ischemic stroke [69]. Catechins may protect the neurons from excess oxidative stress, resulting in the inhibition of neurodegenerative diseases. Misregulated iron metabolism was recently implicated as a central pathological feature in Parkinson’s disease, and thus the iron-chelating properties of epigallocatechin gallate could be important for its protective effects in neurodegenerative diseases [35]. Catechins suppressed morphologic and functional regression in the brain [70] and memory regression in aged mice with accelerated senescence [71]. Although it is not fully elucidated whether catechins pass through the blood–brain barrier or not, epigallocatechin gallate exerted protective effects against neuronal damage after ischemia [58]. Epigallocatechin gallate inhibited β-amyloid formation involved in Alzheimer’s disease, modulated amyloid precursor protein cleavage, and reduced cerebral amyloidosis in mice [72]. A cross-sectional study has revealed that green tea consumption significantly reduced the risk of cognitive dysfunction [73], and was beneficial in Alzheimer’s disease [74,75,76,77]. Moreover, the long-term consumption of tea had an inverse correlation with the onset of Parkinson’s disease in a population study [58,78].

In the central nervous system, senescence and neurodegeneration occur as a consequence of mitochondrial oxidative insults and impaired electron transfer [79]. The accumulation of several oxidation products in neurons during aging prompts the idea that consumption of antioxidant compounds may delay neurodegenerative processes [79]. Thus, catechins may help to prevent neurodegeneration and delay decline of brain function [79].

## 5. The Role of Mitochondria in Oxidative Stress and the Beneficial Effects of Catechins

Mitochondria are central players in the regulation of cell homeostasis. They are essential for energy production but at the same time, reactive oxygen species accumulate as byproducts of the electron transport chain causing mitochondrial damage. Oxidative stress-mediated ROS causes rapid depolarization of mitochondrial inner membrane potential and subsequent impairment of oxidative phosphorylation. Damaged mitochondria produce ROS, especially in the form of the superoxide anion (O^2−^) and hydrogen peroxide (H_2_O_2_), which further accelerate ROS generation [32,80,81]. Opening of mitochondrial permeability transition pore is one of the crucial triggers of apoptosis [82,83]. Furthermore, in cancer cells mitochondrial respiration is suppressed and anaerobic glycolysis, which is known as the Warburg effect, predominates [33,84,85]. In addition, mitochondrial damage is implicated in neurodegenerative diseases [79]. The essential role of mitochondria in oxidative stress imply that they could be also one of the targets of catechins in the cell.

**Figure 3:**
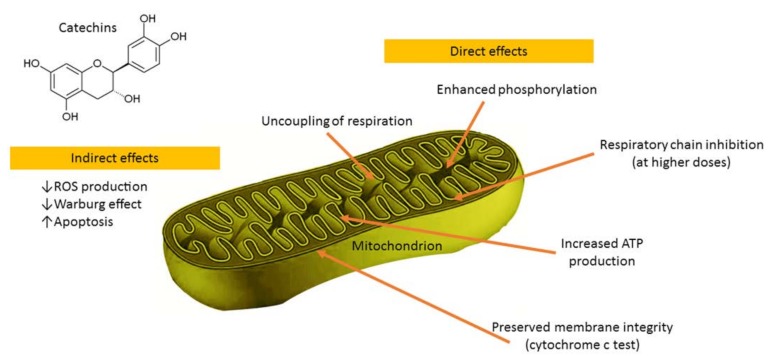
The main effects of catechins on mitochondrial functions.

In primary rat islets cultured with a monomeric catechin-rich cocoa flavanol fraction the increased glucose-stimulated insulin secretion corresponded with enhanced mitochondrial respiration, suggesting improvements in β-cell fuel utilization. Mitochondrial complex III, IV, and V components were up-regulated after culture with the monomer-rich fraction, corresponding with increased cellular ATP production [86]. Epigallocatechin gallate (the main catechin of green tea), at the concentration of 50 μM, increased State 4 complex I-driven respiration, thus demonstrating uncoupling effects on rat liver mitochondria [87]. Furthermore, epigallocatechin gallate increased oxidative phosphorylation and ATP production in both human cultured astrocytes and neurons with different kinetic parameters and without toxicity [88]. Catechin-induced ATP production was only blocked by sodium azide and oligomycin, inhibitors of cytochrome c oxidase (complex IV), and ATP synthase (complex V) respectively [88]. In addition, epigallocatechin gallate (0.3 μM) increased respiratory capacity of neuronal mitochondrial function [89]. Catechin was found to be a potent mitochondrial permeability transition pore inducer over the whole tested concentration range, thus implying its prooxidant action being responsible for this effect [90]. (−)-Epicatechin diminished inhibition of mitochondrial respiration, lowered mitochondrial Ca^2+^ load, and preserved a pool of NADH that correlated with higher tissue ATP levels in the reperfused heart [91]. (−)-Epicatechin stimulated mitochondrial respiration and oxygen consumption in Panc-1 cells [92]. After oral administration in mice, (−)-epicatechin increased respiration of cardiac mitochondria and enhanced free radical production during State 3 respiration [93]. Our group evaluated the direct effects of (−)-epicatechin and procyanidin B2 on the functions of cardiac mitochondria (Figure 3) [94]. The results demonstrated uncoupling of oxidation from phosphorylation, stimulation of phosphorylation at lower concentrations, and inhibition of respiratory chain at higher concentrations as well as (−)-epicatechin-reduced release of cytochrome 𝑐 from mitochondria [94]. These data implicated that the beneficial effects of (−)-epicatechin and its derivatives might be due to direct modulation of mitochondrial functions.

Catechins have the 3-OH group, which interacts with the B-ring through a hydrogen bond, thus placing it practically in the same plane as the A- and C-rings in their molecules [90,95,96]. Such conformation favors an interaction of these compounds with membranes. Since flavonoids are weak acids of a hydrophobic character, it is likely that they could be potentially capable of causing mitochondrial uncoupling [90,97,98]. As the mild mitochondrial uncoupling is implicated to be a highly effective in vivo antioxidant strategy, catechins might be promising candidate pro-drugs alleviating stress-related disturbances of the organism. Moreover, catechins could be widely applied as active compounds for the design and development of novel pharmaceutical and cosmetic products.
